# Real-Time Leak Detection for a Gas Pipeline Using a *k*-NN Classifier and Hybrid AE Features

**DOI:** 10.3390/s21020367

**Published:** 2021-01-07

**Authors:** Thang Bui Quy, Jong-Myon Kim

**Affiliations:** School of Electrical, Electronics, and Computer Engineering, University of Ulsan, Ulsan 44610, Korea; bqthangcndt@gmail.com

**Keywords:** pipeline leak detection, acoustic emission analysis, signal classification, *k*-NN algorithm, hybrid AE features

## Abstract

This paper introduces a technique using a *k*-nearest neighbor (*k*-NN) classifier and hybrid features extracted from acoustic emission (AE) signals for detecting leakages in a gas pipeline. The whole algorithm is embedded in a microcontroller unit (MCU) to detect leaks in real-time. The embedded system receives signals continuously from a sensor mounted on the surface of a gas pipeline to diagnose any leak. To construct the system, AE signals are first recorded from a gas pipeline testbed under various conditions and used to synthesize the leak detection algorithm via offline signal analysis. The current work explores different features of normal/leaking states from corresponding datasets and eliminates redundant and outlier features to improve the performance and guarantee the real-time characteristic of the leak detection program. To obtain the robustness of leak detection, the paper normalizes features and adapts the trained *k*-NN classifier to the specific environment where the system is installed. Aside from using a classifier for categorizing normal/leaking states of a pipeline, the system monitors accumulative leaking event occurrence rate (ALEOR) in conjunction with a defined threshold to conclude the state of the pipeline. The entire proposed system is implemented on the 32F746G-DISCOVERY board, and to verify this system, numerous real AE signals stored in a hard drive are transferred to the board. The experimental results show that the proposed system executes the leak detection algorithm in a period shorter than the total input data time, thus guaranteeing the real-time characteristic. Furthermore, the system always yields high average classification accuracy (ACA) despite adding a white noise to input signal, and false alarms do not occur with a reasonable ALEOR threshold.

## 1. Introduction

Gas pipelines play a vital role in the fuel transportation field. Even though they are designed and assembled according to strict technical principles [1,2], a gas leak could still occur due to material aging and corrosion [3,4], leading to violent explosions causing injuries, human deaths, and pollution of the environment. Hence, a real-time gas pipeline leak detection system is extremely important to reduce catastrophic consequences.

In early times, acoustic emission (AE) was mainly used for detecting growing cracks and discontinuities in materials because it was defined as releasing elastic energies in a deformed material [5]. However, AE is currently referred to as a phenomenon where transient elastic waves are generated by the rapid release of energy from localized sources within a material, or the transient elastic waves so generated [5]. As a result, a leak is also the source of AEs and is detectable with AE equipment. This type of AE source is sometimes called a secondary source to distinguish it from the classic AEs which are caused by material deformation [5]. The AE-based leak detection is therefore feasible. Consequently, many researchers have applied this mechanism to detect a leak in a gas pipeline [6,7,8,9,10,11,12,13]. The leak detection using AE signals is extremely beneficial because it is a non-destructive technique, thus it does not affect the working system [5,14,15,16]. Additionally, the symptom of small leakage is often extraordinarily subtle; for example, a small leak can be caused by an initial crack that does not create an obvious rupture. Hence, AE sensors can be applicable in this case because they offer high sensitivity regarding any early abnormality [17].

Researchers tend to adopt a data-driven approach that trains a classifier using AE features extracted from AE signals to separate pipeline health states to normal or leaking. This approach is appropriate because an AE signal acquired from a gas pipeline is non-stationary [18,19]. Moreover, AE waves attenuate along the pipeline from their emission source to AE sensors [20]; they vary with the environmental conditions of pressure, flow rate, and temperature [21]. Thus, it is challenging to draw an explicit model to identify a leak relying on AE signals exclusively. A classification model learns the leakage manifestation from the supplied training data; hence, it can identify the leak detection problem effectively. However, the computational complexity of existing leak detection methodologies restricts their exploitation in real-time applications, despite the fact that they show high classification accuracy. For example, the Wavelet transform and the signal decomposition algorithms are used to analyze AE signals, and machine learning-based models are used for state classification [10,11,12,22], which can improve accuracy, but their computation is highly complex.

A long gas transportation system usually comprises numerous pipeline segments with diversity in size, shape, and material. Many sensors are spread over that system to monitor the health of different pipeline segments. A wireless-based leak detection system with a server receiving and exploring signals dispatched from remote sensor nodes, as proposed by [23], would not be suitable for AE signal application due to the overload of communication and computation. Therefore, a sensor node should be a smart integrated system that can itself inspect a pipeline segment and report only the health state of pipeline to its server instead of sending a massive amount of AE signals to the server. The advantage of the integration is that it does not require a complex communication network topology between the sensor nodes and the server. Nonetheless, the integrated system must be low-power and compact, because if many devices are installed, they will result in high energy consumption and a bulky system. This is similar to the design presented in [24], which integrated a propane sensor with a low-power system-on-chip device. However, a propane sensor could only detect an obvious gas leak nearby, thus challenging the early gas leakage detection in a large pipeline network, where a tiny leak would occur at any place and any time.

Working from the demand for gas pipeline leak detection and the achievements and limitations of current studies, this work presents a microcontroller unit (MCU)-based system designed to diagnose leakage for a gas pipeline in real-time. The system analyzes AE signals locally to identify a leak and just issues a warning of state changing. Because an MCU-based system only supports a restricted resource in memory and execution speed for computing implementation, the paper exploits a *k*-nearest neighbor (*k*-NN) classifier trained by using hybrid AE features directly extracted from raw AE signals. The *k*-NN algorithm can execute on a limited-resource platform in real-time because it is made up of simple computations and neighbor-searching loops. To optimize the algorithm further, a filtering technique is exploited to remove the least useful elements from the feature pool relying on the three-sigma rule [25] and the Kullback–Leibler (KL) distance [26], which reduces the number of computation cycles and loops in the correspondingly implemented program, thus accelerating the proposed detection system. The selected features are normalized as well; hence, a trained *k*-NN model can be applied to various sensor nodes along a pipeline network. Moreover, the trained model can be updated in run-time to adapt to a sensor installation location or any change in the working conditions.

Prior to implementing the leak detection program on an MCU-based hardware platform, the proposed methodology is offline synthesized using the Matlab 2019a software and AE signal datasets recorded at a gas pipeline testbed under diverse experimental scenarios. Thus, the essential parameters of the *k*-NN classifier (training features and number of nearest neighbors) are chosen to ensure not only the real-time characteristic, but also high accuracy of the leak detection program. Aside from ambient noise, any external factor that can cause the vibrations in the pipeline can trip AE signals. For instance, a random pipe collision triggers a mechanical vibration that generates plentiful elastic waves propagating through the pipeline. AE sensors with enough sensitivity can capture signals resulting from those elastic waves, thus interfering with measured target signals. Hence, a *k*-NN classifier based on AE signals is subjected to discrete events near the testing pipeline, generating false alarms. To address this problem, the current work proposes monitoring the accumulative leaking event occurrence rate (ALEOR) from the output of the state classifier. A final decision of pipeline health state is based on the comparison between the instant ALEOR and a defined threshold, hence avoiding a false alarm.

Finally, the work evaluates the gas pipeline leak detection system constructed from the proposed methodology on the 32F746G-DISCOVERY board (STMicroelectronics, Quakertown, PA, USA) using recorded AE signal datasets. Experimental results demonstrate that the system can identify a leak in real-time with high average classification accuracy under various pressure conditions, and its robustness is satisfactory, even with adding white noise to the input AE signal. Hence, the proposed MCU-based system is applicable for gas leak detection in real applications.

## 2. AE Signal Data Acquisition

**Figure 1:**
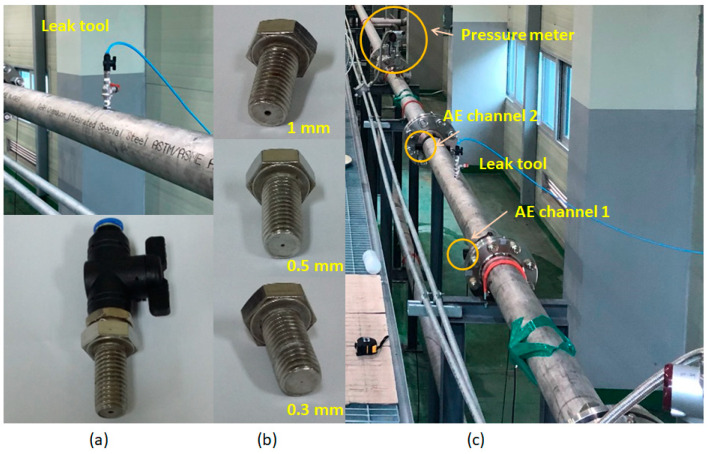
Pipeline testbed: (**a**) leak tool, (**b**) orifices, (**c**) test section.

A pipeline testbed is established to simulate the gas leakage as shown in Figure 1. The testbed is a part of a real gas pipeline system (see Figure 1c) made from stainless steel 304 pipelines with sizes of 114.3 millimeters (mm) and 6.02 mm in outer diameter and wall thickness, respectively. To create various leaks, we designed a leak tool as shown in Figure 1a, which is assembled to the testing pipeline. This tool is composed of a valve and an orifice of diameter 0.3 mm, 0.5 mm, or 1 mm (see Figure 1b). Hence, the normal/leaking states of the pipeline are connected to closed/open valve positions.

**Figure 2:**
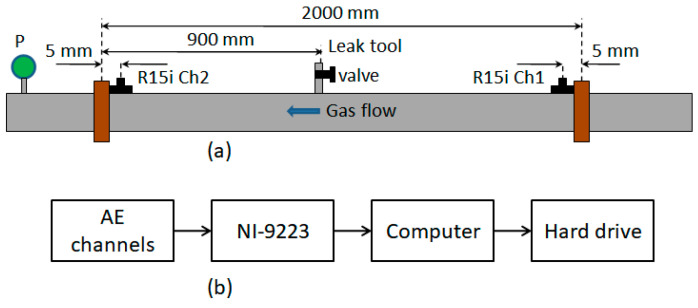
Experimental setup: (**a**) test section, (**b**) data acquisition system. (R15i Ch1 and R15i Ch2 are acoustic emission (AE) channels, P is a pressure meter).

The experimental configuration is shown in Figure 2. To capture AE signals, two R15i-AST sensors (AE channels), which were manufactured by MITRAS Group, Inc (Princeton Junction, NJ, USA), are mounted at downstream and upstream locations on the surface of the testing pipeline. These sensors can detect any elastic wave in a range of operating frequencies, which are 50 kilohertz (kHz) to 400 kHz [27]. Those elastic waves can be caused by diverse sources such as leak noise [10], negative pressure wave [4], ambient noise, and other vibrations of the pipe wall. Such R15i-AST sensors are selected because their operating frequency range covers the frequency ranges of AE waves propagating in metal objects, which are from 100 kHz to 300 kHz, as stated in the BSI standard BS EN 15,856 [15]. AE signals are sampled at 1 megahertz (MHz) by the NI-9223 module. The sampling frequency of 1 MHz is more than double the maximum operating frequency of sensors, thus satisfying the Nyquist–Shannon sampling theorem [28] about converting analog signals into digital signals.

After finishing the hardware setup, data recording software is installed on the computer to control the whole data acquisition. Additionally, we exploit the pencil lead break technique [29] to examine both sensitivity of sensors and the whole AE equipment. This ensures the reliability of AE signal datasets prior to storing them in the hard drive.

**Figure 3:**
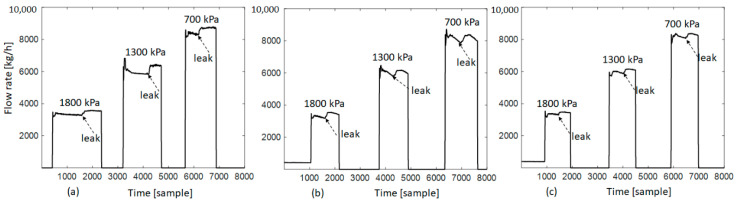
Gas flow rates corresponding to three orifices: (**a**) 0.3 mm, (**b**) 0.5 mm, (**c**) 1.0 mm.

In the experiment, the three orifices are alternated to simulate different leakages at three inner relative pressures of 700 kPa, 1300 kPa, and 1800 kPa, resulting in three normal states of the testing pipeline (closed valve) and nine diverse leaking states (open valve). Specifically, data acquisition has been performed as follows. First, an orifice was installed, and the pipeline system was configured at a pressure level of 700 kPa, 1300 kPa, or 1800 kPa, and this condition was kept relatively stable before acquiring AE signals. At this time, the valve of the leak tool was closed to simulate the normal state of the pipeline. For this state, the signals were recorded for 2 min. Next, the valve was opened to simulate a leakage. Here, the data corresponding to a leaking state were collected after pressure stabilization. Figure 3 presents gas flow rates measured in front of the testing pipeline during the experimental stages.

## 3. Leak Detection Methodology

**Figure 4:**
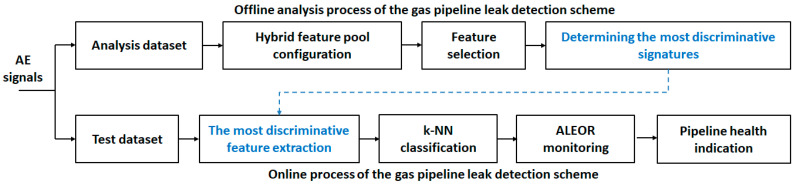
Entire flow diagram of the gas pipeline leak detection.

The overall gas pipeline leak detection diagram is shown in Figure 4. It is composed of two processes: one is offline, and the other is online. The offline analysis synthesizes and optimizes the leak detection algorithm, while the online process experiments and verifies the detection. We will describe the analysis blocks of the algorithm below.

### 3.1. Hybrid Feature Pool and Feature Selection

To detect the leaking state of a gas pipeline, time and frequency domain statistical features are extracted, as shown in Table 1, from raw AE signals utilized as diagnosis leakage signatures. We therefore obtain a hybrid feature pool of size *R* × *M*, where *R* is the number of feature types (*R* = 12, as shown in Table 1), and *M* is the number of analyzed signal frames. The value *M* should be large enough to reflect the statistical discrimination of the normal/leaking states precisely.

Next, the feature pool should be refined to enhance the pipeline health classification quality. Outliers, data points that differ significantly from the other aggregated data points in the same class can cause serious problems in statistical analyses. The existence of outliers in a feature extracted from an AE signal measured at a gas pipeline is inevitable, resulting from both exterior and interior factors. The exterior factor could be variability in the measurement. For example, power spikes can interfere with sensed signals, causing outliers in AE features. This problem can be fixed by perfect experimental configuration and the exploitation of high-quality equipment. Outliers may be created by interior factors of the pipeline system, such as burst emissions appearing in high amplitude and energy in AE signals. A gas pipeline itself generates such a signal due to the disturbance between inner gas flow and the gas flow–pipe wall interaction. Nevertheless, outliers should be eliminated from features used for training a classifier because they do not statistically characterize the normal/leaking state discrimination, thus leading to the deterioration of the classification performance. This paper assumes a normal distribution for the AE features; outliers can therefore be detected by the three-sigma rule [25]. This rule is expressed as follows:

(1) $$\mathrm{Pr}\left(\left|Y_{i}-\mu_{y_{i}}\right|\leq 3\sigma_{y_{i}}\right)\approx 0.99.$$

where *Y_i_* is an observation from a normally distributed feature *y_i_*; *μ_yi_* and *σ_yi_* are the mean and standard deviation of the distribution, respectively; *i* = 1, 2, …, *R*. According to (1), if |*Y_i_* − *μ_yi_*| > 3*σ_yi_*, the value *Y_i_* is considered an outlier and it is removed from the set of *y_i_*-feature observations. After unwanted observations are eliminated from the *y_i_*-vector, the length of *y_i_*-vector is shrunk as M_i_* (M_i_* ≤ *M*). Because the feature types distribute dissimilarly, the outlier elimination might return different lengths M_i_* of the *y_i_*-vectors (*i* = 1, 2, …, *R*). As a result, we compensate new satisfactory observations for the feature pool to gain M_i_* = *M*. The feature pool size is therefore intact (*R* × *M*); however, its elements are refined, which satisfies (1).

Furthermore, all the extracted features may not be equally effective in highly accurate leak detection. Inferior signatures not only impair the classification accuracy but also increase the computational complexity. Thus, we need to filter out redundant features from the pool to enhance the detection performance while reducing the computational load. This paper scores features using the Kullback–Leibler distance [26] and eradicates low-ranked elements in the feature pool. The KL distance is calculated as follows:

(2) $$\begin{array}{l} d_{KL}=D_{12}+D_{21};\\ D_{12}=\sum p\left(y_{i}|w_{1}\right)\ln\frac{p\left(y_{i}|w_{1}\right)}{p\left(y_{i}|w_{2}\right)};D_{21}=\sum p\left(y_{i}|w_{2}\right)\ln\frac{p\left(y_{i}|w_{2}\right)}{p\left(y_{i}|w_{1}\right)}. \end{array}$$

where *d_KL_* is the KL distance, *w*_1_, *w*_2_ are two classes indicating the normal and leaking states, respectively; *y_i_* = [*y_i_*_1_, *y_i_*_2_, …, *y_iM_*]^T^ is a sort of *y_i_*-feature in the refined feature pool, *p* is a conditional probability density function. Based on (2), we retain features with the dominant KL distance and remove the others in the feature pool, because the greater the KL distance is, the more discriminative the feature. Finally, we retrieve a purified feature pool with size *r* × *M*, where *r* is the number of high-scored features (*r* ≤ *R*).

### 3.2. Leak Detection Using a k-NN Classifier and Accumulative Leaking Event Occurrence Rate

With the purified feature pool, we utilize a *k*-NN classifier to distinguish the two normal/leaking states, in which an obscure new class is assigned to the most common class among its *k* nearest neighbors using the Manhattan distance given by:

(3) $$\delta_{j}=\sum_{n=1}^{r}\left|z_{n}-y_{n,j}\right|.$$

where *δ_j_* is the Manhattan distance between the input feature vector *z* = {*z*_1_, *z*_2_, …, *z_r_*} and the *j*th training feature vector y_*j_ = {*y*_1*j*_, *y*_2*j*_, …, *y_rj_*}, and *j* = 1, 2, …, *M*. The *k*-NN classifier categorizes the input z into the major class in its *k* nearest neighbors corresponding to *k* minimum distances *δ_j_* (*k* < *M*).

The detection approach aims at the extremely noisy industrial environment. A *k*-NN classifier is sensitive to noise involving ambient noise and discrete events and may subsequently yield a false alarm (the classified state is “leaking” but the true state is “normal”) or miss a true leaking event (the leakage is actually happening); thus, a normal/leaking state decision should depend on monitoring the ALEOR. The leak detection criterion is given by:

(4) $$ALEOR=\frac{\Delta B}{\Delta t}\geq \gamma ,\;\Delta t=t_{2}-t_{1}.$$

where Δ*B* is the number of leaking events in a time period Δ*t* = *t*_2_ − *t*_1_, which is from the moment t1 to the moment *t*_2_, and *γ* is a threshold to issue a warning of pipeline health state. This threshold is flexibly adjusted by pipeline operators in their specific real environment.

## 4. Implementation of Proposed Gas Pipeline Leak Detection on an MCU-Based Architecture

### 4.1. Offline Analysis of AE Signal Datasets

Prior to developing the real-time gas leak detection program with the proposed methodology on an MCU-based architecture, we analyzed offline AE signal datasets to search for a set of optimal parameters, thus enhancing the performance of the real-time leak detection program. The optimized parameters are the feature pool for training the *k*-NN classification model and the number *k* (the number of nearest neighbors used for the *k*-NN classifier). We perform the offline analysis process using a number of AE datasets, as shown in Table 2.

For feature selection, we should first normalize extracted features to place them on the same unit basis. The feature normalization is expressed by the following equation:

(5) $$y_{new}=\frac{y_{old}-\mu_{yn}}{\sigma_{yn}}\;.$$

where *y_old_*, *y_new_* are original and rescaled features, respectively, and *µ_yn_*, *σ_yn_* are successively mean and standard deviation of the feature estimated from samples belonging to the normal pipeline state.

**Figure 5:**
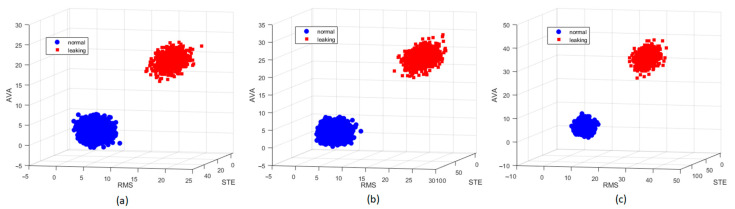
Three-dimensional visualization of the three most highly ranked features under various pressure conditions: (**a**) P_0_, (**b**) P_1_, (**c**) P_2_.

Table 3 exhibits feature scores using the KL distance method. The most highly ranked features are STE, RMS, AVA, and STD, and these are returned in every pressure condition. Hence, we only consider these kinds of features to build the real-time gas leak detection program. Figure 5 illustrates the 3-D visualization of three features with the highest scores under diverse pressure conditions, in which the normal/leaking states are obviously separated for all the cases. Moreover, we know that a large *k* may improve performance; however, too large a *k* destroys the locality. Therefore, to choose *k* appropriately, we employ the available *k*-NN fitting function “fitcknn” supported by Matlab 2019a to trial different values of *k* using the analysis datasets and we obtain *k* = 25.

The datasets belong to a signal channel (R15i Ch1 or R15i Ch2), corresponding to three pressure conditions: 700 kPa (P_0_), 1300 kPa (P_1_), and 1800 kPa (P_2_), and pipeline health states: normal (L_0_), leaking (0.3 mm (L_1_), 0.5 mm (L_2_), and 1 mm (L_3_)), which were recorded in Section 2; N_FA_ and N_FE_ are the numbers of frames for the offline analysis and experiment respectively, and a frame consists of 8192 samples stored in the hard drive.

### 4.2. Gas Pipeline Leak Detection Implementation on an MCU-Based Hardware Architecture

#### 4.2.1. Overview of the Experimental Hardware Design with an MCU Used for Real-Time Gas Pipeline Leak Detection

**Figure 6:**
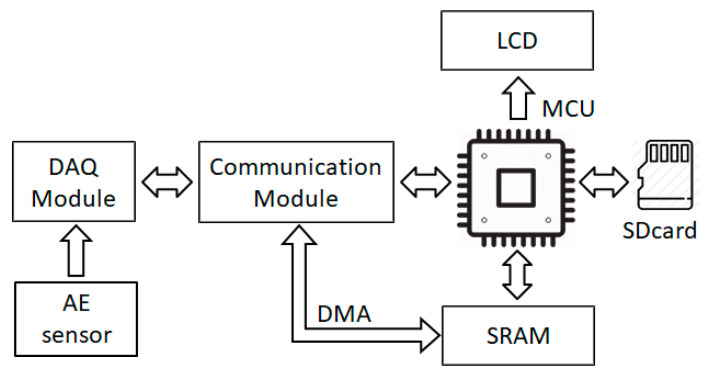
Experimental MCU-based hardware architecture for the gas pipeline leak detection.

**Figure 7:**
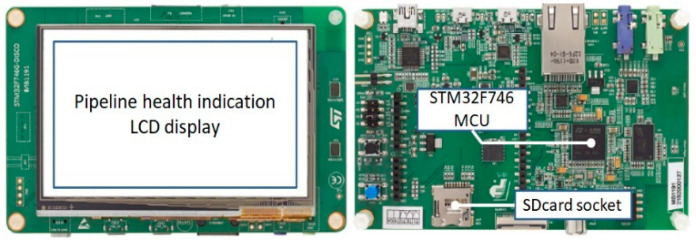
32F746G-DISCOVERY board (tope view: left side—bottom view: right side).

Figure 6 illustrates an MCU-based hardware architecture to implement the proposed method for real-time gas pipeline leak detection. A sensor channel is connected to a data acquisition (DAQ) module which converts analog AE signals to digital AE signals and directly writes them to a synchronous random-access memory (SRAM) through a communication module, along with a direct memory access (DMA) channel available in the MCU; hence, the leak detection program can investigate AE signals in real-time. We also design a portable memory (SDcard) to store some pre-defined parameters of the leak detection program and its runtime log files used for later analyses. Hence, the program can be adjusted and updated quickly. Additionally, a liquid crystal display (LCD) is installed to indicate the output of the diagnostic program. This entire experimental design is embedded in the 32F746G-DISCOVERY board, as shown in Figure 7.

#### 4.2.2. Real-Time Gas Leak Detection Implementation on the 32F746G-DISCOVERY Board

Due to the limitation of the MCU in internal memory and operating speed, we use integer instead of floating-point format for the feature calculation and the *k*-NN classification, thus utilizing the memory economically and lightening the computation load. In other words, a real feature value is multiplied by 10 before rounding it, which sustains a one-decimal point precision for the vectors of rounded features, while avoiding reduction in the classification quality.

**Figure 8:**
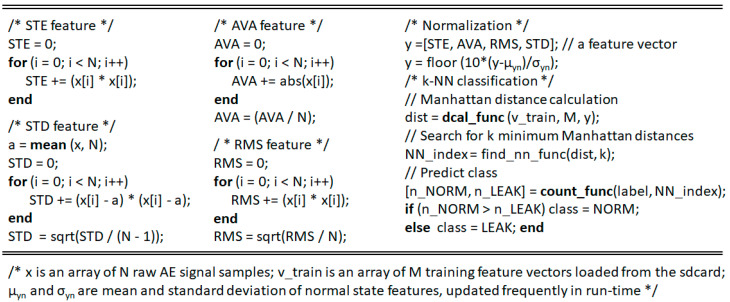
Primary program module of real-time gas pipeline leak detection embedded in the 32F746G-DISCOVERY board.

A trained classifier leans heavily on its training datasets, while AE signals acquired from a pipeline are prone to variation because the inner flow rate and pressure change constantly. The signals also fluctuate according to the sensor installation location and the operating moment. To reconcile these differing environments, we must adjust the trained leak detection model to its real and specific operational conditions. Therefore, the paper proposes updating the classifier by modifying the two parameters *µ_yn_* and *σ_yn_* related to the normal pipeline state in run-time, and which are employed in (5). Figure 8 shows the feature calculation and *k*-NN classification module of a real-time gas pipeline leak detection program implemented on the 32F746G-DISCOVERY board.

## 5. Experimental Results

To evaluate the gas pipeline leak detection system quickly, we emulate a real data acquisition device (DAQ) using a computer program which dispatches recorded AE signal datasets, whose description is shown in Table 2, through an available communication channel to the 32F746G-DISCOVERY board. This does not affect the objective assessment because the datasets have been acquired from a practical pipeline testbed under various operating conditions. We here figure out three aspects: detection accuracy, real-time characteristic, and detection robustness, because those are key factors to apply a leak detection system for the real environment.

### 5.1. Detection Accuracy and Real-Time Characteristic

**Figure 9:**
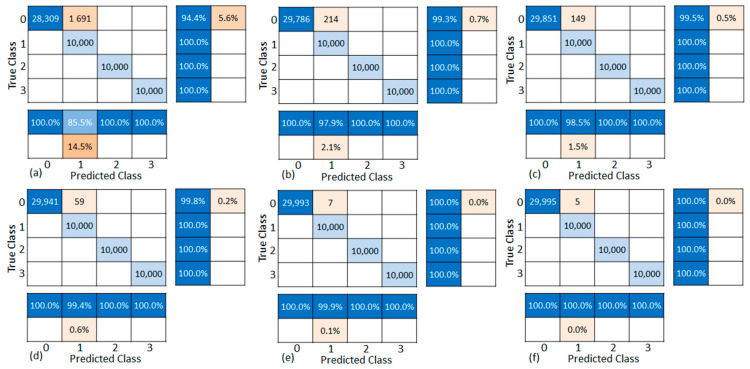
Confusion matrices resulting from experimental scenarios: R15i Ch1 (**a**) P_0_, (**b**) P_1_, (**c**) P_2_; R15i Ch2 (**d**) P_0_, (**e**) P_1_, (**f**) P_2_ (classes 0, 1, 2, 3, and 4 are L_0_, L_1_, L_2_, and L_3_, respectively).

**Figure 10:**
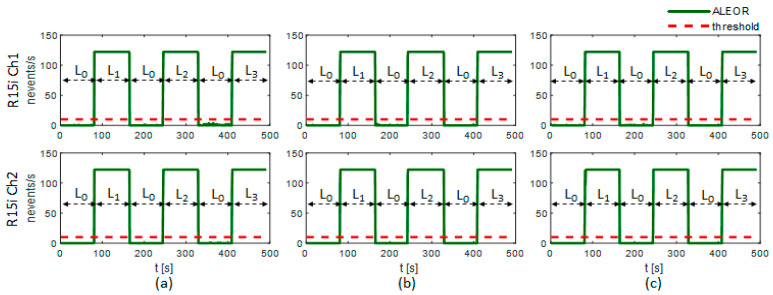
ALEOR under different pressure conditions: (**a**) P_0_, (**b**) P_1_, (**c**) P_2_.

Figure 9 shows confusion matrices of experimental results returned by the leak detection program running on the 32F746G-DISCOVERY board, and Table 4 illustrates classification accuracy and execution time for evaluation scenarios. The accuracy, as averaged over the two sensor channels (R15i Ch1 and Ch2), and that of various pipeline states (L_0_, L_1_, L_2_, and L_3_), is relatively high at better than 98% for every pressure condition (P_0_, P_1_, and P_2_). Besides, the mean execution time (t_E_ = 109 s) is less than the total experimental dataset duration (t_D_ = 123 s). This demonstrates the real-time characteristic of the implemented detection system that does not miss any data and returns a timely result during the analysis operation. Furthermore, the ALEOR is monitored while examining dataset pairs (L_0_, L_1_), (L_0_, L_2_), and (L_0_, L_3_) subsequently (see Figure 10). This plot reveals the correct identification of pipeline states: normal (L_0_), leaking (L_1_, L_2_, and L_3_), exploiting a threshold *γ* = 10 (see red dash line in Figure 10). The leaking state is decided only if ALEOR exceeds the threshold, despite the fluctuation below it. Therefore, no false alarm is reported in the experiment and the leaking state is also indicated punctually.

### 5.2. Detection Robustness

The result as exhibited in Table 4 and Figure 9 and Figure 10 is obtained by using the test datasets under the same recording condition as the training datasets. As a result, the effectiveness of the proposed leak detection system may not be adequately demonstrated, because in a real gas pipeline network, there are always irregular disturbances leading to AE signal modifications, such as operating mode variation (inner pressure or flow rate), noise interference, etc. Measurement of an AE sensor can be modelled as follows:

(6) z=x+η

where *z* and *x* are measured and original signals, respectively, and *η* represents any signal modification including ambient noise and discrete events. We assume the normal distribution function for both *x* and *η*. According to the probability rule specified by [30], *z* distributes normally also, and its mean and standard deviation are sequentially:

(7) $$\mu_{z}=\mu_{x}+\mu_{\eta };\;\sigma_{z}=\sqrt{\sigma_{x}^{2}+\sigma_{\eta }^{2}}$$

where *μ_z_*, *σ_z_*, *μ_x_*, *σ_x_*, *μ_η_*, *σ_η_* are means and standard deviations of *z*, *x*, and *η*, respectively. Equation (7) shows that the abnormal disturbance distorts original signals, thus deteriorating the signal-based leak detection model.

**Figure 11:**
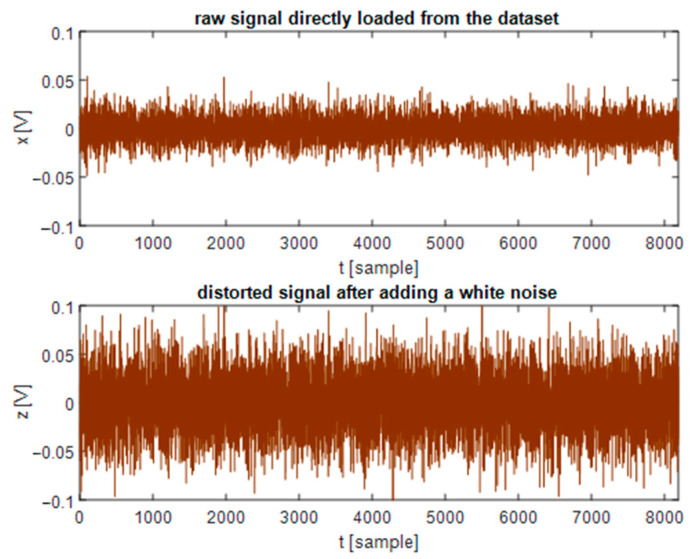
A signal after adding a white noise with *ρ* = 2.

To verify the robustness of the proposed leak detection method, we add white noise to the experimental datasets prior to conducting the real-time leak detection on the 32F746G-DISCOVERY board. This noise is referred to as the signal disturbance *η*, simulated by an available function in the Matlab software with a rule below:

(8) $$\mu_{\eta }=0;\;\sigma_{\eta }=\rho \times \sigma_{xn}$$

where *σ_xn_* is the standard deviation of normal state signal (acquired when the pipeline is healthy), and *ρ* is a proportion ratio. We set *μ_η_* = 0 in (8) because the mean parameter of a signal is mainly related to low frequency components of that signal, while the operating frequency range of R15i sensors is from 50 kHz to 400 kHz. The low frequency band (below 50 kHz) is not examined and the influence of *μ_η_* is therefore relatively minor or *μ_η_* ≈ 0. Figure 11 illustrates the signal distortion if adding a white noise *η* according to (6) and (8) where *ρ* = 2. We can easily realize that the distorted signal energy is greater than the original because of the added noise in Figure 11.

**Figure 12:**
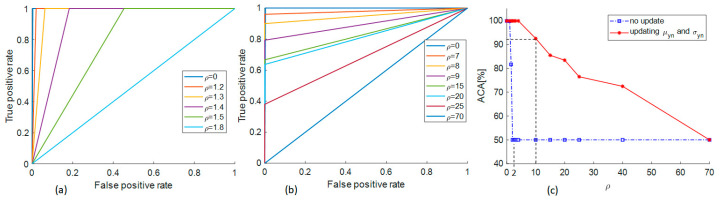
ROC and ACA according to *ρ*: (**a**) ROC without updating *µ_yn_* and *σ_yn_*, (**b**) ROC with updating *µ_yn_* and *σ_yn_*, (**c**) ACA reduction.

**Figure 13:**
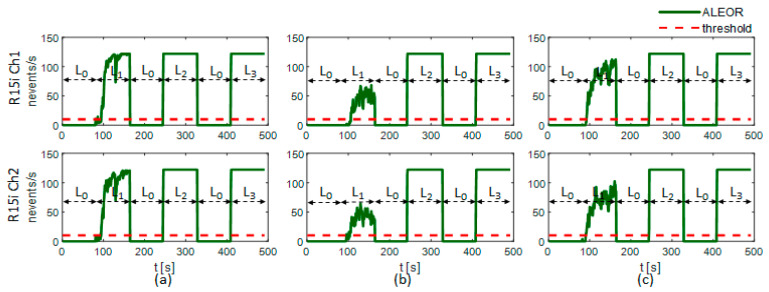
ALEOR under different pressure conditions: (**a**) P_0_, (**b**) P_1_, (**c**) P_2_ (After adding a white noise with *ρ* = 10).

We alter *ρ* and observe the performance deterioration of the trained classifier. Figure 12 shows the dependence of receiver operating characteristic (ROC) and average classification accuracy (ACA) on *ρ*. The computation is calculated on all the datasets of the two sensor channels in two cases: with updating *μ_yn_* and *σ_yn_* (see Section 4.2.2) and without updating. The classification performance substantially declines at slight values of *ρ* if we do not adapt the model to the increasing added white noise (see Figure 12a and the blue dash dot line in Figure 12c). In contrast, the classifier can still work acceptably until *ρ* = 70 if we adjust *μ_yn_* and *σ_yn_* (see Figure 12b and the red solid line in Figure 12c). With *ρ* = 10, the resulting classification accuracy is above 90% (see Figure 12c) and the pipeline state can be exactly identified by the ALEOR with a threshold *γ* = 10, as shown in Figure 13 for every experimental condition. In short, the proposed methodology can ensure the robustness of the leak detection system.

Although the proposed method can sustain a high classification performance with small values of *ρ*, the classification performance still deteriorates gradually according to the increase in *ρ* and the classifier cannot precisely operate with *ρ* > 70 which causes severe distortion of the acquired signals. Therefore, we should configure the testbed to resemble an applied real pipeline before gathering datasets for training the classifier, thus obtaining an adequate leakage detector. The greater the similarity between the testbed and the real pipeline, the more accurate the detection is.

## 6. Conclusions

A complete system is offered for real-time gas pipeline leak detection in the paper. First, the system offline analyzed recorded AE signals sampled at 1 MHz. The process configured a hybrid feature pool and normalized its elements using the mean and standard deviation of the set of feature observations related to normal pipeline health. Then, the pool was purified using the three-sigma rule and the Kullback–Leibler distance to obtain the most discriminative signatures. Next, the system identified the pipeline health states (normal/leaking) with an input vector of features, by exploiting a *k*-nearest neighbor classifier that seeks the purified feature pool for the signatures closest to the input vector, based on the Manhattan distance. To avoid issuing a false alarm, the system decided a pipeline state via monitoring the accumulative leaking event occurrence rate and a predefined threshold. Finally, the total proposed leak detection method was embedded in a compact MCU-based hardware platform for real-time leak detection. The detection accuracy, the real-time characteristic, and the robustness of the introduced gas pipeline leak detection system have been evaluated. The experimental results showed that the system indicated pipeline health states robustly in a quick enough timeframe for real-time application. Thus, this system can be applied for inspecting pipeline health in a real gas pipeline network.

The testbed used in this paper for collecting AE signals is a part of a real gas pipeline network. Hence, the resulting AE signals are not simple signals generated by the pipeline leakage simulation in a laboratory. They do not only contain information about pipeline states (normal or leaking), but also depend on practical gas transportation and systematic behavior. Additionally, a noisy measurement location and wave attenuation could conceal symptoms of leakage in recorded signals. This challenges the signal investigation because the relation between the leakage phenomenon and AE signals is unclear in the initial analysis stages. Therefore, a short pipeline was chosen in the paper to easily separate signal classes related to pipeline states corresponding to different experimental scenarios, hence conveniently proposing a leak detection method as well as evaluating experimental results. However, it is believed that the proposed technique can effectively monitor a long pipeline in a real application. The pipeline length depends on the signal detection ability of the AE sensor—their sensitivity and a specific working environment. These parameters can be estimated by using pencil lead breaking tests.

## Figures and Tables

**Table 1:** Typical features for leak detection.

Features	Equations	Features	Equations	Features	Equations
Short time energy (STE)	$\sum_{n=0}^{N-1}x_{n}^{2}$	Standard deviation (STD, σ)	$\sqrt{\frac{1}{N-1}{\sum_{n=0}^{N-1}\left(x_{n}-\mu \right)}^{2}}$	Skewness (SKE)	$\frac{1}{N}{\sum_{n=0}^{N-1}\left(\frac{x_{n}-\mu }{\sigma }\right)}^{3}$
Root mean square (RMS)	$\sqrt{\frac{1}{N}\sum_{n=0}^{N-1}x_{n}^{2}}$	Zero crossing rate (ZCR)	$\frac{1}{N}\sum_{n=1}^{N-1}\left|\mathrm{sign}\left(x_{n}-\mu \right)-\mathrm{sign}\left(x_{n-1}-\mu \right)\right|$	Spectral peak (SPP)	$\underset{f}{\arg\max}\left(X\left(f\right)\right)$
Average amplitude (AVA)	$\frac{1}{N}\sum_{n=0}^{N-1}\left|x_{n}\right|$	Entropy (ETY)	$-\sum_{n=0}^{N-1}q_{n}\log_{2}q_{n}$	Spectral centroid (SPC, $f_{c}$)	$\frac{\sum_{m=0}^{M-1}f_{m}X_{m}^{2}}{\sum_{m=0}^{M-1}X_{m}^{2}}$
Mean (MEA, µ)	$\frac{1}{N}\sum_{n=0}^{N-1}x_{n}$	Kurtosis (KUS)	$\frac{1}{N}{\sum_{n=0}^{N-1}\left(\frac{x_{n}-\mu }{\sigma }\right)}^{4}$	Spectral spread (SPS)	$\sqrt{\frac{\sum_{m=0}^{M-1}{\left(f_{m}-f_{c}\right)}^{2}X_{m}^{2}}{\sum_{m=0}^{M-1}X_{m}^{2}}}$

Where *x* is an input signal, *N* is the total number of samples, *X* is the short-time spectral amplitude, *f* is the frequency, *M* is the total number of discrete frequencies, and $q_{n}=x_{n}^{2}/\sum_{n=0}^{N-1}x_{n}^{2}$.

**Table 2:** Number of datasets used for the offline analysis and evaluation.

	P_0_		P_1_		P_2_	
	N_FA_	N_FE_	N_FA_	N_FE_	N_FA_	N_FE_
L_0_	600	30,000	600	30,000	600	30,000
L_1_	200	10,000	200	10,000	200	10,000
L_2_	200	10,000	200	10,000	200	10,000
L_3_	200	10,000	200	10,000	200	10,000

**Table 3:** Feature score based on KL distance.

	STE	RMS	AVA	MEA	STD	ZCR	ETY	KUS	SKE	SPP	SPC	SPS
P_0_	57.7	36.5	37.3	−30.2	36.2	10.8	4.0	6.9	−18.0	1.8	7.0	8.9
P_1_	71.9	44.2	44.4	−0.2	44.0	10.2	−1.7	3.3	−9.1	2.0	7.8	7.6
P_2_	77.7	47.2	47.4	5.1	47.1	13.3	−5.6	1.1	−6.5	3.3	11.1	9.0

**Table 4:** Classification accuracy and execution time.

		P_0_			P_1_			P_2_		
		A	t_D_	t_E_	A	t_D_	t_E_	A	t_D_	t_E_
R15i Ch1	L_0_	97.2	246	214	99.7	246	214	99.8	246	214
	L_1_	92.8	82	74	99.0	82	74	99.3	82	74
	L_2_	100	82	74	100	82	74	100	82	74
	L_3_	100	82	74	100	82	74	100	82	74
R15i Ch2	L_0_	99.9	246	214	100	246	214	100	246	214
	L_1_	99.7	82	74	100	82	74	100	82	74
	L_2_	100	82	74	100	82	74	100	82	74
	L_3_	100	82	74	100	82	74	100	82	74
Average		98.7	123	109	99.8	123	109	99.9	123	109

Where t_D_ and t_E_ are the total time of datasets and execution time, respectively, measured in seconds. A is classification accuracy given by: A = 100 × N_C_/N_FE_ [%], N_C_ is the number of correctly classified frames.

## Data Availability

Data sharing not applicable.
